# Na–Ga–Si type-I clathrate single crystals grown *via* Na evaporation using Na–Ga and Na–Ga–Sn fluxes†

**DOI:** 10.1039/c8ra07971d

**Published:** 2018-12-04

**Authors:** Hironao Urushiyama, Haruhiko Morito, Hisanori Yamane, Masami Terauchi

**Affiliations:** Institute of Multidisciplinary Research for Advanced Materials, Tohoku University 2-1-1 Katahira, Aoba-ku Sendai 980-8577 Japan; Institute for Materials Research, Tohoku University 2-1-1 Katahira, Aoba-ku Sendai 980-8577 Japan morito@imr.tohoku.ac.jp

## Abstract

Single crystals of a Na–Ga–Si clathrate, Na_8_Ga_5.70_Si_40.30_, of size 2.9 mm were grown *via* the evaporation of Na from a Na–Ga–Si melt with the molar ratio of Na : Ga : Si = 4 : 1 : 2 at 773 K for 21 h under an Ar atmosphere. The crystal structure was analyzed using X-ray diffraction with the model of the type-I clathrate (cubic, *a* = 10.3266(2) Å, space group *Pm*3̄*n*, no. 223). By adding Sn to a Na–Ga–Si melt (Na : Ga : Si : Sn = 6 : 1 : 2 : 1), single crystals of Na_8_Ga_*x*_Si_46−*x*_ (*x* = 4.94–5.52, *a* = 10.3020(2)–10.3210(3) Å), with the maximum size of 3.7 mm, were obtained *via* Na evaporation at 723–873 K. The electrical resistivities of Na_8_Ga_5.70_Si_40.30_ and Na_8_Ga_4.94_Si_41.06_ were 1.40 and 0.72 mΩ cm, respectively, at 300 K, and metallic temperature dependences of the resistivities were observed. In the Si L_2,3_ soft X-ray emission spectrum of Na_8_Ga_5.70_Si_40.30_, a weak peak originating from the lowest conduction band in the undoped Si_46_ was observed at an emission energy of 98 eV.

## Abstract 2

Single crystals of a Na–Ga–Si clathrate, Na_8_Ga_4.94_Si_41.06_, of size 3.7 mm were grown *via* the evaporation of Na from a Na–Ga–Si–Sn melt with the molar ratio of Na : Ga : Si : Sn = 6 : 1 : 2 : 1 at 873 K for 3 h under an Ar atmosphere.

## Graphical abstract

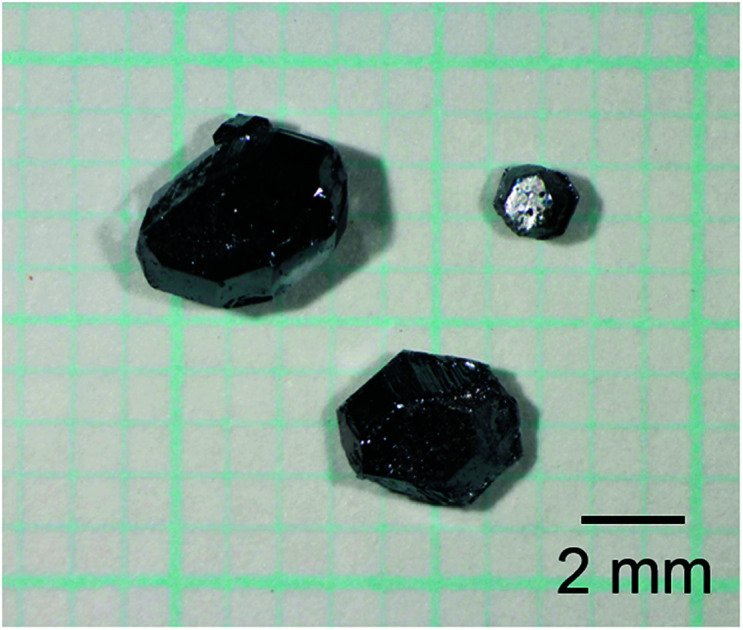

## 1. Introduction

Na_8_Si_46_ type-I clathrate has an open framework structure composed of dodecahedral [Si]_20_ cages with five-membered rings of Si [5^12^] and tetrakaidecahedral [Si]_24_ cages with five-membered rings and six-membered rings of Si [5^12^6^2^].^1^ Each cage encapsulates a Na as a guest atom. Since this compound was first synthesized by Kasper *et al.* in 1965,^1^ it has attracted significant attention from researchers owing to its unique structure as well as being a member of group 14 intermetallic chathrates, the variants of which are of interest for photovoltaic,^2^ thermoelectric,^3^ and superconducting materials.^4^

Na_8_Si_46_ has conventionally been synthesized along with another type of Na–Si clathrate (type-II, Na_24_Si_36_) *via* the solid-thermal decomposition of Na_4_Si_4_ (melting point 1071 K (ref. 5)) in the temperature range 593–823 K under high vacuum (<10^−2^ Pa).^1,6,7^ The samples obtained by this method are powdery solids with a grain size of micrometers. The Na–Si clathrates have been regarded as metastable or intermediate phases. The single crystals of Na_8_Si_46_ could not be directly grown from the Na–Si melt. In 2009, Beekman *et al.* succeeded in growing type-II Na_24_Si_136_ single crystal growth using a spark plasma sintering (SPS) system.^8^ Single crystals of type-I and type-II clathrates were selectively synthesized by the reaction of Na_4_Si_4_ and graphite flakes with a spatial separation layer of NaCl.^9,10^ This process was named the kinetically controlled thermal decomposition (KCTD) method. Type-I Na_8_Si_46_ single crystal with sizes of about 200 μm was obtained by heating at 858 K.^9^ Recently, our research group found that the single crystal of the Na–Si binary clathrates can be grown in Na–Sn rich Na–Sn–Si ternary melt by Na evaporation.^11,12^ Single crystals of type-I and type-II clathrates were selectively prepared by setting the growth temperatures at 773 and 873 K, respectively. The maximum size of the type-I Na_8_Si_46_ single crystal was 5 × 3 × 3 mm^3^, having {110} habit planes.^12^

The electric properties of ternary type-I clathrates A_8_M_8_Si_38_ (A = Na, K, Rb, Cs; M = Al, Ga, In) have been predicted using first-principles calculation.^13,14^ Indirect transition semiconductor characters of A_8_M_8_Si_38_ with band gaps of 0.45–0.89 eV^13^ and their thermoelectric properties^14^ were presented by the calculation. A_8_Al_8_Si_38_ (A = K, Rb, Cs) have been synthesized using flux materials of Al and alkali-metal halide salts.^15^ The samples for the transport measurements were prepared by compacting at 4 GPa in a high-pressure multianvil apparatus. Dong *et al.* synthesized microcrystalline Na_8_Al_8_Si_38_ by the KCTD method using a Na_4_Si_4_ + NaAlSi mixture as the precursor and prepared bulk polycrystalline samples by SPS to characterize the transport properties.^16^ Sui *et al.* synthesized A_8_Ga_8_Si_38_ (A = K, Rb, Cs) *via* a direct reaction of constituent elements.^17^ They sintered the powders of A_8_Ga_8_Si_38_ using SPS to obtain polycrystalline bulk samples for characterization of thermoelectric properties, and measured the band gaps of 1.14–1.18 eV by the surface electromotive force of the powder samples.^17^ A cubic lattice parameter of 10.36 Å was only reported for Na_8_Ga_23_Si_23_,^18^ but details of synthesis and crystal structures of the clathrates have not been clarified. The present paper reports crystal growth of the type-I clathrate Na_8_Ga_5.70_Si_40.30_ using a Na–Ga–Si melt and Na_8_Ga_*x*_Si_46−*x*_ (*x* = 4.94–5.52) using Na–Ga–Si–Sn melts. The crystal structures were analyzed by single crystal X-ray diffraction (XRD) and some single crystals were characterized by electrical resistivity measurement and soft X-ray emission spectroscopy (SXES).

## 2. Experimental

As starting raw materials, Na (Nippon Soda Co., Ltd., purity 99.95%), Si powder (Kojundo Chemical Laboratory Co., Ltd., 4N), granular Ga (Dowa Electronics Co., 6N), and granular Sn (Mitsuwa Chemicals Co., Ltd., 5N) were used. These raw materials were weighed in a molar ratio of Na : Ga : Si = 4 : 1 : 2 (total 12.19 mmol) or Na : Ga : Si : Sn = 6 : 1 : 2 : 1 (total 8.70 mmol) in an Ar-filled glove box. They were placed in a boron nitride crucible (Showa Denko Co., Ltd., purity 99.95%, outer diameter 8.5 mm, inner diameter 6.5 mm, depth 18 mm) and sealed in a stainless-steel container (SUS 316: outer diameter 12.7 mm, inner diameter 10.7 mm, height 80 mm) together with Ar gas. The crucible was heated in an electric furnace at 1123 K for 12 h, and thereafter cooled to room temperature to prepare the starting sample of Na–Ga–Si or Na–Ga–Si–Sn.

The crucible containing the starting sample was taken out from the stainless-steel container in the glove box under Ar atmosphere, and transferred to the upper part of another long stainless-steel container (outer diameter 12.7 mm, inner diameter 10.7 mm, height 300 mm). A schematic view of the container is shown in Fig. S1 of ESI.† The crucible at the upper part of the container was heated at 723–873 K for 3–24 h in atmospheric-pressure Ar using a tubular electric furnace, and the lower part of the container was cooled with a fan to maintain a temperature gradient in the container. Na evaporated from the melt of the starting sample was condensed at the lower inside part of the container. After heating, the sample was cooled to room temperature by turning off the electric power to the furnace and taken out the sample from the container in the glove box. The amount of evaporated Na was evaluated using the weight loss of the sample after heating. Unvaporized Na and Na–Ga, Na–Si, and/or Na–Sn compounds formed in the sample were reacted with 2-propanol and ethanol in air, and subsequently, the water-soluble reactants were removed by washing with water. Single crystals were obtained after the removal of residual Ga and Sn *via* a reaction with hydrochloric acid water solution (35.0–37.0 mass% HCl) (alcohol and acid treatments).

**Caution**: confirm the complete decomposition of the reactive compounds containing Na through reaction with alcohol before washing with water.

The compositions of the single crystals were analyzed using an electron probe microanalyzer (EPMA, JEOL, JXA-8200) attached to wavelength dispersive X-ray spectrometers. The densities of the single crystals were measured using the Archimedes method. The X-ray diffraction (XRD) data of the single crystals were collected using a single-crystal XRD diffractometer (Bruker, D8QUEST, Mo-Kα radiation) and analyzed using the APEX3 program.^19^ X-ray absorption correction and structure refinement were performed by using the SADABS^19^ and SHELEXL-97 programs,^20^ respectively. The structures of Si/Ga cages containing Na were depicted using VESTA.^21^ Soft X-ray emission (SXE) spectra were measured using an SXE spectrometer attached to a port of a wavelength-dispersive spectrometer of a scanning electron microscope (SEM, JEOL, JSM-6480LV^22,23^). The electric resistivities of the single crystals were measured from 10 to 300 K using the four-terminal method with Ag paste electrodes.

## 3. Results and discussion

**Fig. 1:**
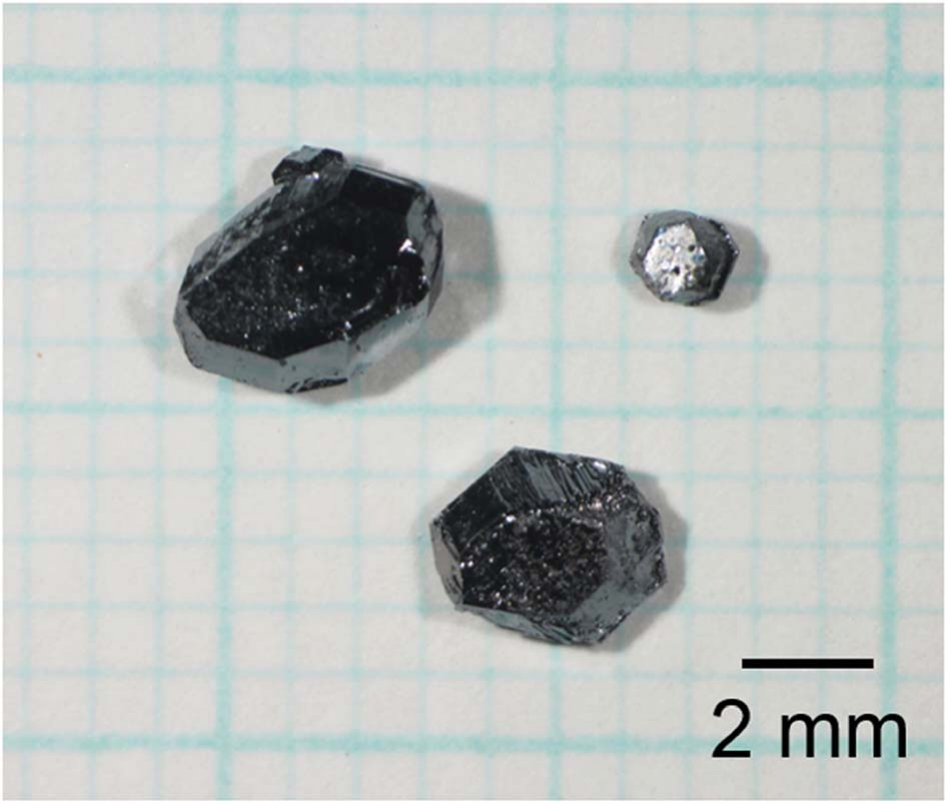
Optical micrographs of the single crystals grown *via* Na evaporation from the Na–Ga–Si–Sn starting sample at 873 K for 3 h under an Ar atmosphere (∼10^5^ Pa), followed by alcohol and acid treatments.

The conditions of crystal growth and mole fractions of the evaporated Na against the initial amounts of Na in the starting samples are listed in Table 1. It can be observed that 84% of Na was evaporated during the heating of Na–Ga–Si starting sample at 773 K for 21 h. Single crystals of size up to 2.9 mm were obtained. When the Na–Ga–Si–Sn starting samples were heated at 723 K for 24 h, 773 K for 12 h, 823 K for 9 h, and 873 K for 3 h, 48–55% of Na was evaporated. Fig. 1 shows an optical micrograph of the single crystals grown by heating the Na–Ga–Si–Sn starting sample at 873 K for 3 h. The maximum sizes of the single crystals grown at 723, 773, 823, and 873 K were 2.3, 2.5, 2.6, and 3.7 mm, respectively.

**Table 1:** Compositions of the starting samples, heating conditions, mole fractions of the evaporated Na from the starting samples, and the maximum sizes of the obtained crystals

Sample	Composition of the starting sample	Temp. (K)	Time (h)	Evaporated Na (mol mol^−1^)	Crystal size (mm)
**Na** **:** **Ga** **:** **Si**					
1	4 : 1 : 2	773	21	0.84	2.9

**Na** **:** **Ga** **:** **Si** **:** **Sn**					
2	6 : 1 : 2 : 1	723	24	0.49	2.3
3	6 : 1 : 2 : 1	773	12	0.50	2.5
4	6 : 1 : 2 : 1	823	9	0.48	2.6
5	6 : 1 : 2 : 1	873	3	0.55	3.7

**Fig. 2:**
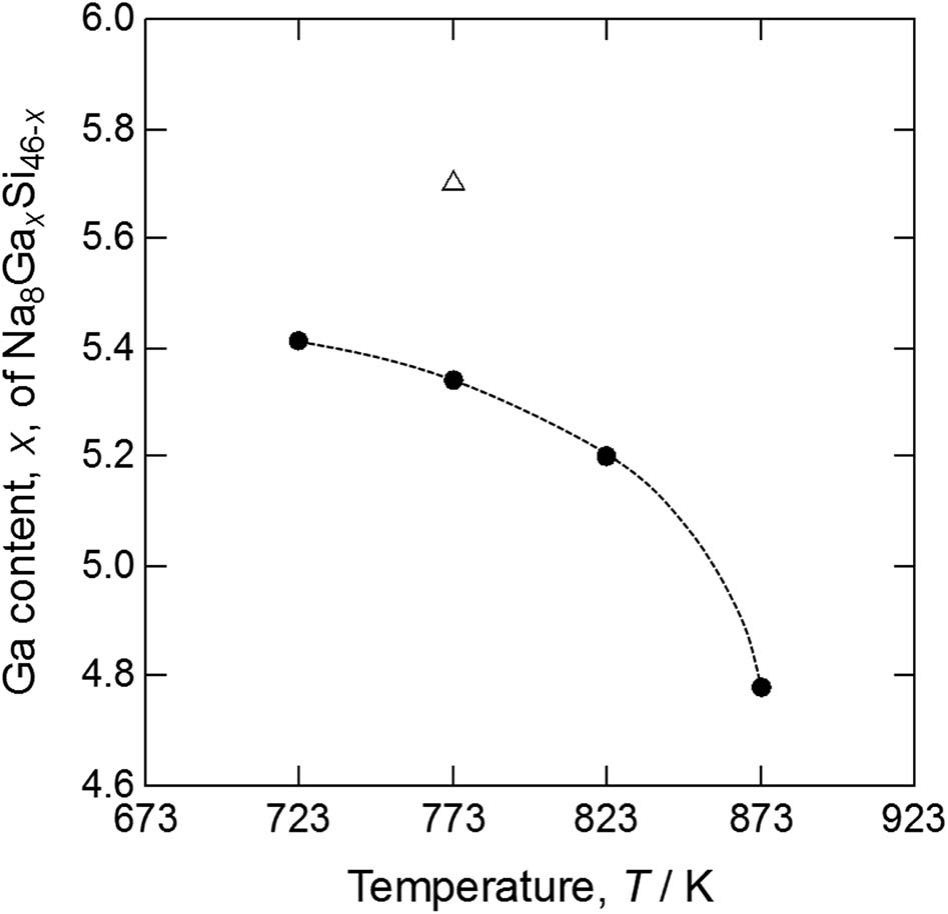
Ga content of type-I clathrates Na_8_Ga_*x*_Si_46−*x*_*versus* heating temperatures of Na–Ga–Si mixture (△) and Na–Ga–Si–Sn mixtures (●).

Table 2 shows the compositions of the single crystals grown by heating the Na–Ga–Si starting sample at 773 K (crystal 1) and the Na–Ga–Si–Sn starting samples at 723 (crystal 2), 773 (crystal 3), 823 (crystal 4), and 873 K (crystal 5). Elements other than Na, Ga, and Si were not detected from these crystals using EPMA. When the total number of the Ga and Si cage atoms was set to 46, based on the ideal formula of type-I clathrates Na_8_Ga_*x*_Si_46−*x*_, the contents of Na analyzed using EPMA were converted from 7.99(6) to 8.23(6), which is close to the ideal Na number of 8. The Ga number of crystal 1 grown at 773 K from the Na–Ga–Si starting sample was 5.67(8). The Ga numbers *x* of Na_8_Ga_*x*_Si_46−*x*_ for crystals 2, 3, 4, and 5 grown from the Na–Ga–Si–Sn starting mixture were 5.41(3), 5.35(3), 5.19(6), and 4.78(3) respectively, which decreased with the increase in the heating temperature from 723 to 873 K as shown in Fig. 2.

**Table 2:** Chemical compositions analyzed by EPMA for crystals 1–5

	Chemical composition			
	Na (at%)	Ga (at%)	Si (at%)	Chemical formula based on Si + Ga = 46
1	14.91(10)	10.48(15)	74.6(2)	Na_8.06(6)_Ga_5.67(8)_Si_40.33(13)_
2	14.99(12)	10.00(6)	75.01(12)	Na_8.11(6)_Ga_5.41(3)_Si_40.59(6)_
3	15.03(5)	9.88(5)	75.09(9)	Na_8.14(3)_Ga_5.35(3)_Si_40.65(5)_
4	15.18(12)	9.58(12)	75.3(2)	Na_8.23(6)_Ga_5.19(6)_Si_40.81(12)_
5	14.79(11)	8.85(6)	76.36(17)	Na_7.99(6)_Ga_4.78(3)_Si_41.22(9)_

**Fig. 3:**
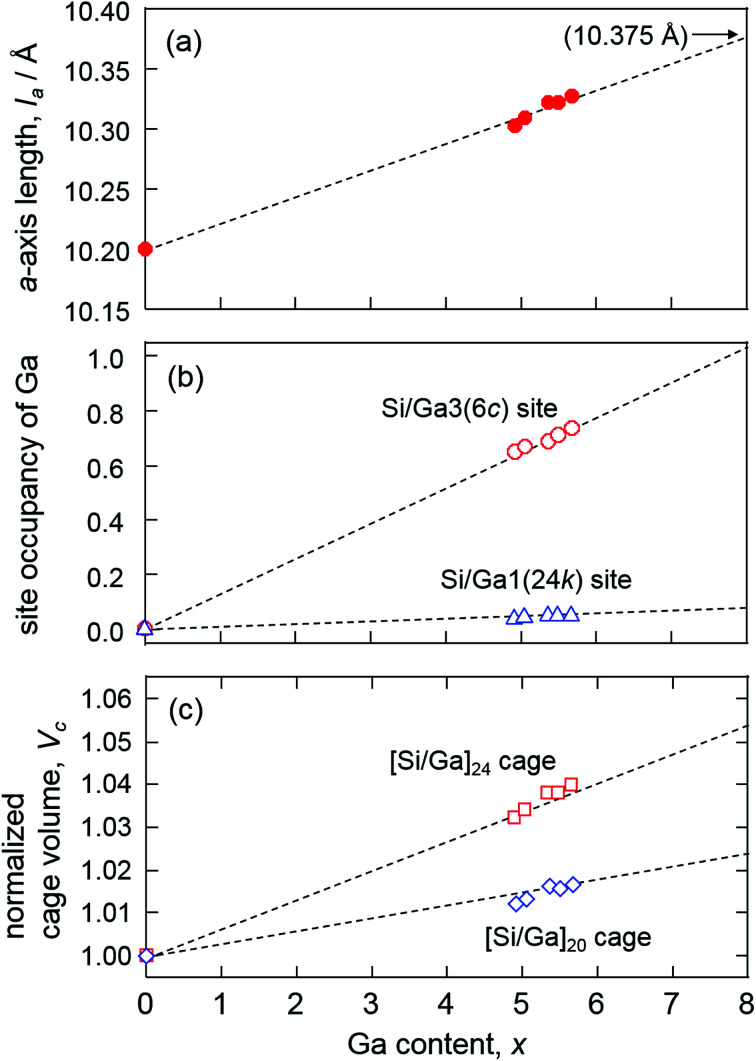
Plots of *a*-axis length (a), 6c and 24k site occupancies of Ga (b), and normalized volumes of [Si/Ga]_24_ cage and [Si/Ga]_20_ cage (c) *versus* Ga content *x* of Na_8_Ga_*x*_Si_46−*x*_.

The results of crystal structure analysis and refined atomic positional and equivalent isotropic displacement parameters of the single crystals are shown in Tables 3 and S1.† All the single crystals were analyzed with the type-I structure (cubic system, space group *Pm*3̄*n*). In the structure refinements, Na1(6d) and Na2(2a) sites were fully occupied and the occupancies of Ga in Si/Ga1(24k), Si2(16i), and Si/Ga3(6c) sites were refined. As the occupancy of the Si2(16i) site equaled 1 within the standard deviation, it was fixed to 1 in the final refinements. The reliability factors *R*_1_ (all data) for samples 1–5 were 1.02–1.90%. The formulae of the clathrates based on the refined occupancies were in accordance with those determined using EPMA. The *a*-axis length of Na_8_Ga_*x*_Si_46−*x*_ increased from 10.3020(2) to 10.3226(2) Å with the increase in the Ga content *x* from 4.94(6) to 5.70(7) as shown in Fig. 3(a). The densities in the range 2.554–2.584 Mg m^−3^, which were calculated using the lattice parameters and the formulas, were consistent with those measured using the Archimedes method (Table 3).

**Table 3:** Crystal data, data collection and refinement in X-ray single crystal diffraction analysis for crystals 1–5^a^

Crystal	1	2	3	4	5
Chemical formula	Na_8_Ga_5.70(7)_Si_40.30_	Na_8_Ga_5.52(7)_Si_40.48_	Na_8_Ga_5.39(11)_Si_40.61_	Na_8_Ga_5.06(7)_Si_40.94_	Na_8_Ga_4.94(6)_Si_41.06_
Formula weight, *M*_r_ (g mol^−1^)	1713.35	1705.86	1700.37	1686.71	1681.71
Temperature, *T* (K)	298(2)	302(2)	302(2)	300(2)	300(2)
Crystal system	Cubic	Cubic	Cubic	Cubic	Cubic
Space group	*Pm*3̄*n*	*Pm*3̄*n*	*Pm*3̄*n*	*Pm*3̄*n*	*Pm*3̄*n*
Unit-cell dimensions, *a* (Å)	10.3266(2)	10.3210(3)	10.3210(4)	10.3090(2)	10.3020(2)
Unit-cell volume, *V* (Å^3^)	1101.21(4)	1099.42(6)	1099.42(7)	1095.59(4)	1093.36(6)
*Z*	1	1	1	1	1
Measured density, *D*_obs_ (Mg m^−3^)	2.584(1)	2.561(9)	2.563(2)	2.541(5)	2.514(5)
Calculated density, *D*_cal_ (Mg m^−3^)	2.584	2.576	2.568	2.556	2.554
Radiation wavelength, *l* (Å)	0.71073	0.71073	0.71073	0.71073	0.71073
Size (mm)	0.125 × 0.177 × 0.188	0.163 × 0.126 × 0.124	0.207 × 0.130 × 0.124	0.246 × 0.214 ×0.168	0.253 × 0.193 × 0.153
Absorption correction	Multi-scan	Multi-scan	Multi-scan	Multi-scan	Multi-scan
Absorption coefficient, *μ* (mm^−1^)	4.635	4.540	4.464	4.291	4.231
Limiting indices	−9 ≤ *h* ≤ 11	−5 ≤ *h* ≤ 13	−13 ≤ *h* ≤ 10	−11 ≤ *h* ≤ 12	−6 ≤ *h* ≤ 13
	−13 ≤ *k* ≤ 9	−12 ≤ *k* ≤ 8	−11 ≤ *k* ≤ 11	−13 ≤ *k* ≤ 6	−11 ≤ *k* ≤ 11
	−13 ≤ *l* ≤ 11	−10 ≤ *l* ≤ 13	−13 ≤ *l* ≤ 6	−9 ≤ *l* ≤ 13	−12 ≤ *l* ≤ 13
*F*_000_	829	826	824	818	816
*θ* range for date collection, °	2.790–27.414	2.791–27.430	2.791–27.430	2.794–27.464	2.796–27.484
Reflections collected/unique	2738/252	2594/250	2618/250	2586/249	2581/250
*R*_int_	0.0234	0.0264	0.0292	0.0263	0.0265
Date/restraints/parameters	250/0/18	250/0/18	250/0/18	249/0/18	250/0/18
Weight parameters, *a*, *b*	0.0164, 0.2950	0.0141, 0.1813	0.0187, 0.7742	0.0151, 0.1907	0.0110, 0.2230
Goodness-of-fit on *F*^2^, S	1.153	1.188	1.248	1.232	1.296
*R*_1_, w*R*_2_ (*I* > 2s(*I*))	0.0118, 0.0304	0.0120, 0.0307	0.0176, 0.0427	0.0106, 0.0299	0.0100, 0.0269
*R*_1_, w*R*_2_ (all data)	0.0125, 0.0309	0.0127, 0.0311	0.0190, 0.0442	0.0108, 0.0300	0.0102, 0.0269
Largest diff. peak and hole, Δ*r* (e Å^−3^)	0.300, −0.244	0.298, −0.176	0.374, −0.292	0.244, −0.133	0.381, −0.200

a *R*_1_ = Σ||*F*_o_| − |*F*_c_||/Σ|*F*_o_|. w*R*_2_ = [Σ*w*(*F*_o_^2^ − *F*_c_^2^)^2^/Σ(*wF*_o_^2^)^2^]^1/2^, *w* = 1/[*σ*^2^(*F*_o_^2^) + (*aP*)^2^ + *bP*], where *F*_o_ is the observed structure factor, *F*_c_ is the calculated structure factor, *σ* is the standard deviation of *F*_c_^2^, and *P* = (*F*_o_^2^ + 2*F*_c_^2^)/3. *S* = [Σ*w*(*F*_o_^2^ − *F*_c_^2^)^2^/(*n* − *p*)]^1/2^, where *n* is the number of reflections and *p* is the total number of parameters refined.

**Fig. 4:**
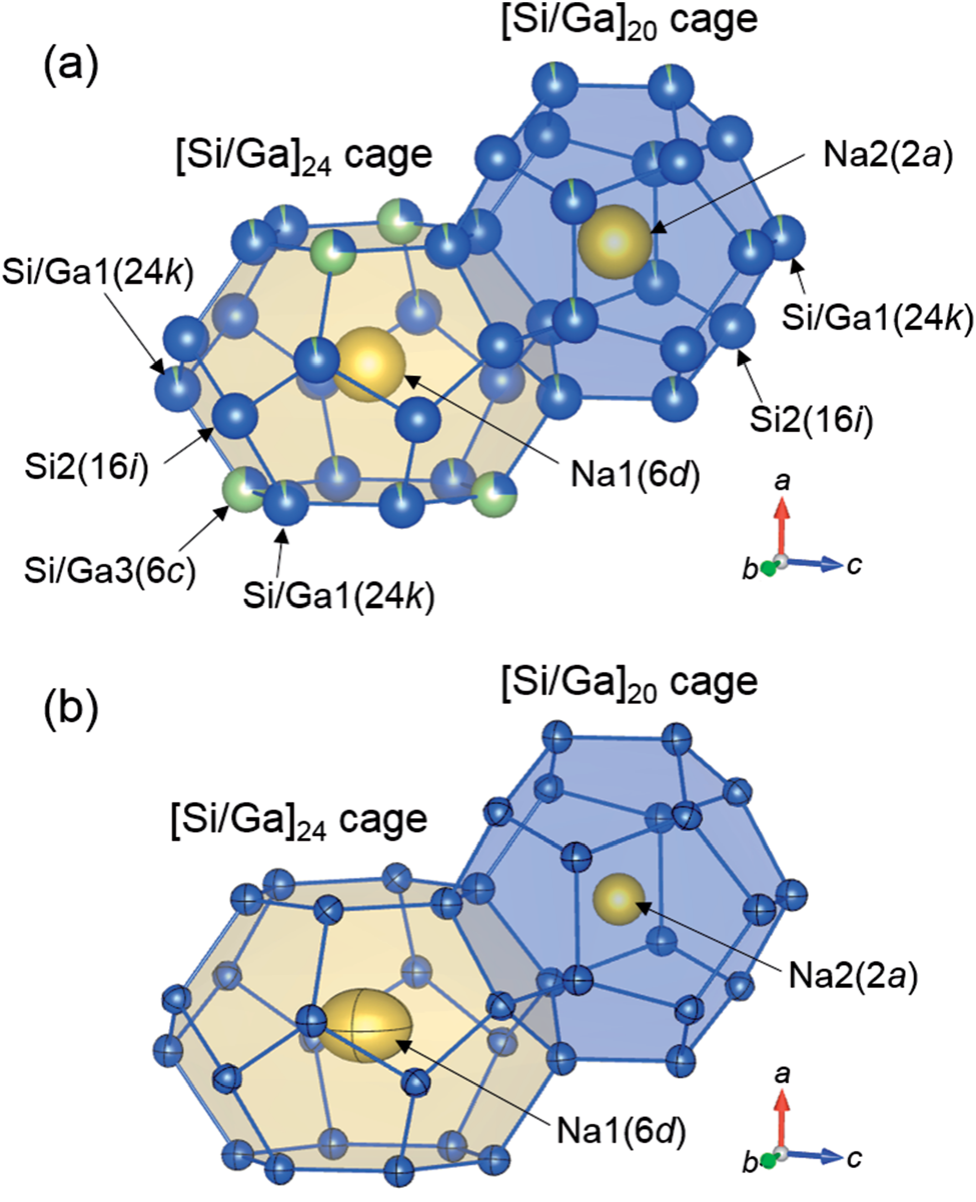
[Si/Ga]_20_ and [Si/Ga]_24_ cages of Na_8_Ga_5.70_Si_40.30_ depicting with spheres of occupancies (a) and 99% probability displacement ellipsoids (b).

The Na2-centered [Si/Ga]_20_ and Na1-centered [Si/Ga]_24_ cages of Na_8_Ga_5.70_Si_40.30_ are shown in Fig. 4(a). The occupancies of Ga in the Si/Ga1(24k) and Si/Ga3(6c) sites are plotted against the Ga content *x* in Fig. 3(b). The Si/Ga3 site, constituting six-membered rings of the [Si/Ga]_24_ cage, was preferentially occupied by Ga atoms with occupancies in the range 64.8(3)–73.0(4)%. The volume increasing rate of the [Si/Ga]_24_ cage is larger than that of the [Si/Ga]_20_ cage (Fig. 3(c)). Similar Ga preferential occupation of the Si/Ga3 site in the [Si/Ga]_24_ cages, which prevents Ga–Ga atom contact, was previously reported for A_8_Ga_8_Si_38_ (A = K, Rb, Cs) type-I clathrate.^17^

In Fig. 4(b), the atomic arrangement of Na_8_Ga_5.70_Si_40.30_ is shown with 99% probability ellipsoids using the anisotropic atomic displacement parameters (Table S2†). The ellipsoids of the Si and Si/Ga sites are small and close to spherical. The atomic displacement parameters of the Na2 site at the center of the [Si/Ga]_20_ regular dodecahedron (*U*_11_ = *U*_22_ = *U*_33_: 0.0229(5)–0.2038(6) Å^2^) are approximately four times larger than those of the Si and Si/Ga sites. The parameters of *U*_22_ = *U*_33_(0.0715(9) − 0.0787(11) Å^2^) for the Na1 site in the [Si/Ga]_24_ cages are the largest in each structure, indicating large displacement of Na atoms at the Na1 site in the direction parallel to the six-membered ring of the Si/Ga1 and Si/Ga3 sites.

**Fig. 5:**
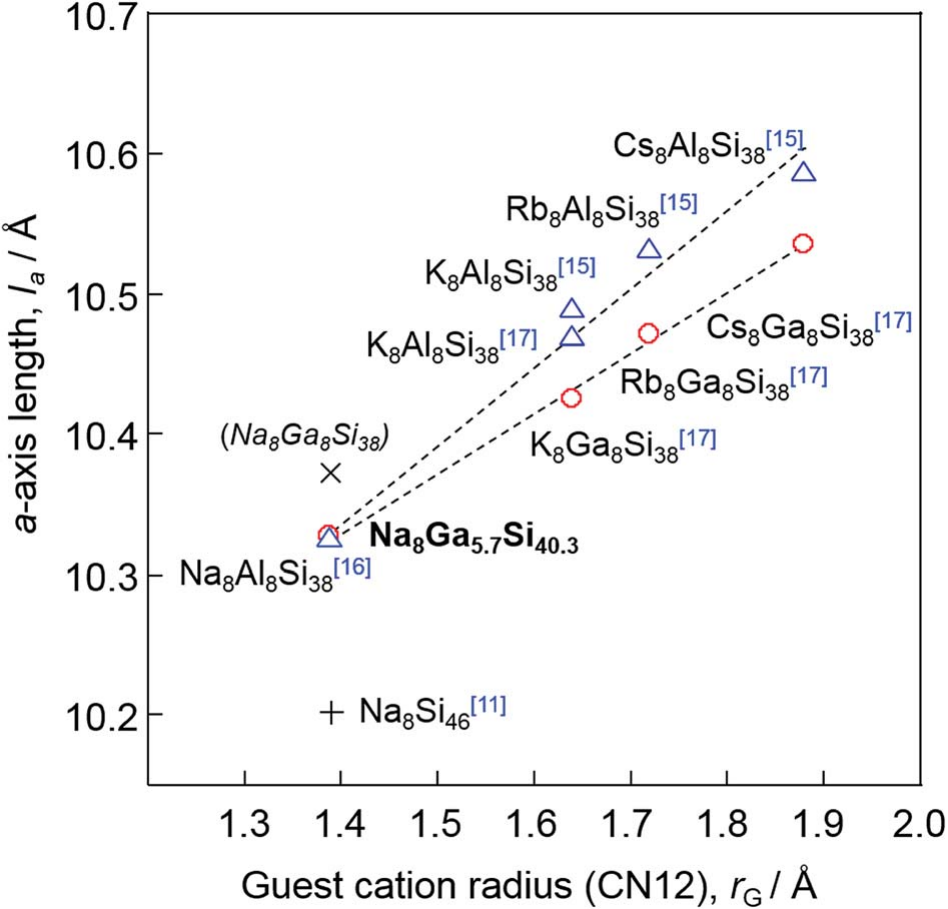
Plot of *a*-axis length *versus* ionic radius (coordination number 12) of the guest atoms for Na_8_Si_46_,^11^ Na_8_Ga_5.7_Si_40.3_ and A_8_(Al/Ga)_8_Si_38_ (A = Na, K, Rb, and Cs).^15–17^ The hypothetical *a*-axis length of Na_8_Ga_8_Si_38_ estimated in Fig. 3(a) is also plotted with ×.

Dong *et al.* reported the preparation of Na_8_Al_8_Si_38_ using SPS and a linear relationship between the *a*-axis lengths of A_8_Al_8_Si_38_ (A = Na, K, Rb, Cs, Ba) and the ionic radii (coordination number CN = 12) of A.^16^ In the present study, the highest Ga content *x* of Na_8_Ga_*x*_Si_46−*x*_ was 5.70(7), whereas A_8_Ga_8_Si_38_ (*x* = 8) was reported for other alkali elements A = K, Rb, and Cs.^17^ However, the *a*-axis lengths of Na_8_Ga_5.70_Si_40.30_ and these type-I gallium silicon clathrates are plotted on the same line against the ionic radii of the alkali metals as shown in Fig. 5. The *a*-axis lengths of A_8_Ga_8_Si_38_, A = K, Rb, and Cs, are smaller than the corresponding values of A_8_Al_8_Si_38_, but the lengths of Na_8_Al_8_Si_38_ (10.3260(1) Å) and Na_8_Ga_5.70_Si_40.30_ (10.3266(2) Å) are similar. As there is a linear relation between the *a*-axis length of Na_8_Ga_*x*_Si_46−*x*_ and *x*, the *a*-axis length of hypothetical Na_8_Ga_8_Si_36_ could be estimated to be 10.375 Å *via* extrapolation (Fig. 3(a)). The length is plotted out of the line in the graph shown in Fig. 5. Although it is not apparent whether Na_8_Ga_8_Si_36_ was formed at this moment, there might be an upper size limit of cages containing Na atoms in the type-I clathrate structure. Thus, a high-pressure condition may be required to crystalize Na_8_Ga_8_Si_38_.

**Fig. 6:**
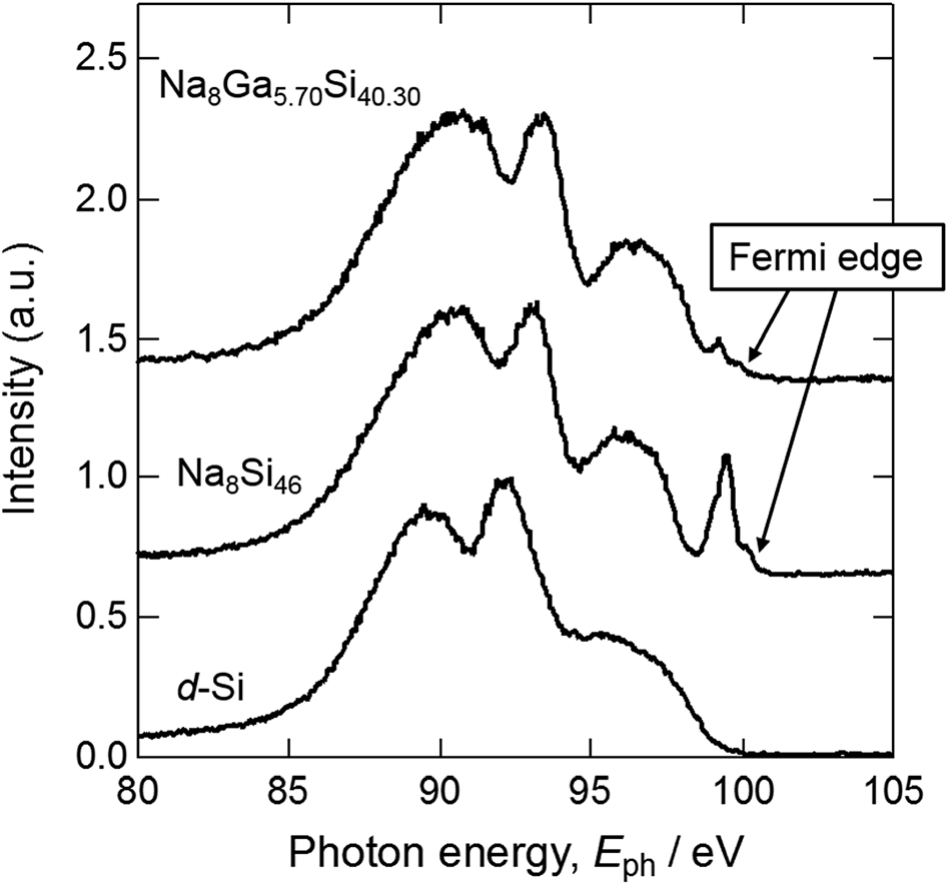
Si L_2,3_ SXE spectra of Na_8_Ga_5.70_Si_40.30_, Na_8_Si_46_, and d-Si.

Fig. 6 shows the SXE spectra of crystal 1 (Na_8_Ga_5.70_Si_40.30_), Na–Si binary type-I clathrate (Na_8_Si_46_) synthesized in the previous study,^11^ and diamond-type cubic Si (d-Si), which was the same as the starting material. The peaks observed around 99 and 98 eV in the spectra of Na_8_Si_46_ and Na_8_Ga_5.70_Si_40.30_ are consistent with the peak observed at 99 eV for Na_8_Si_46_ by Moewes *et al.*^24^ It was considered that the peak edges corresponded to the lower end of the conduction band where the Fermi level exists. Although the peak of 99 eV with a sharp edge at 101 eV was observed for Na_8_Si_46_, the corresponding peak of Na_8_Ga_5.70_Si_40.30_ was small, which indicates the difference between the electronic states of these clathrates.

The peak observed around 93–94 eV in the Na_8_Si_46_ spectrum shifted higher by approximately 0.5 eV from that of the d-Si. The peak of Na_8_Ga_5.70_Si_40.30_ was further shifted by approximately +0.3 eV from that of Na_8_Si_46_. The Si–Si distances of d-Si and Na_8_Si_46_, and the Si/Ga–Si/Ga distance of Na_8_Ga_5.70_Si_40.30_ were 2.35166 Å,^25^ 2.3286(11)–2.3941(9) Å,^11^ and 2.3610(10)–2.4370(4) Å (Table S3†), respectively. As the Si–Si or Si/Ga–Si/Ga distance increases, the band gap decreases. Consequently, the energy gaps between the inner shell levels and valence band increase and the spectra shift to the higher energy side.

**Fig. 7:**
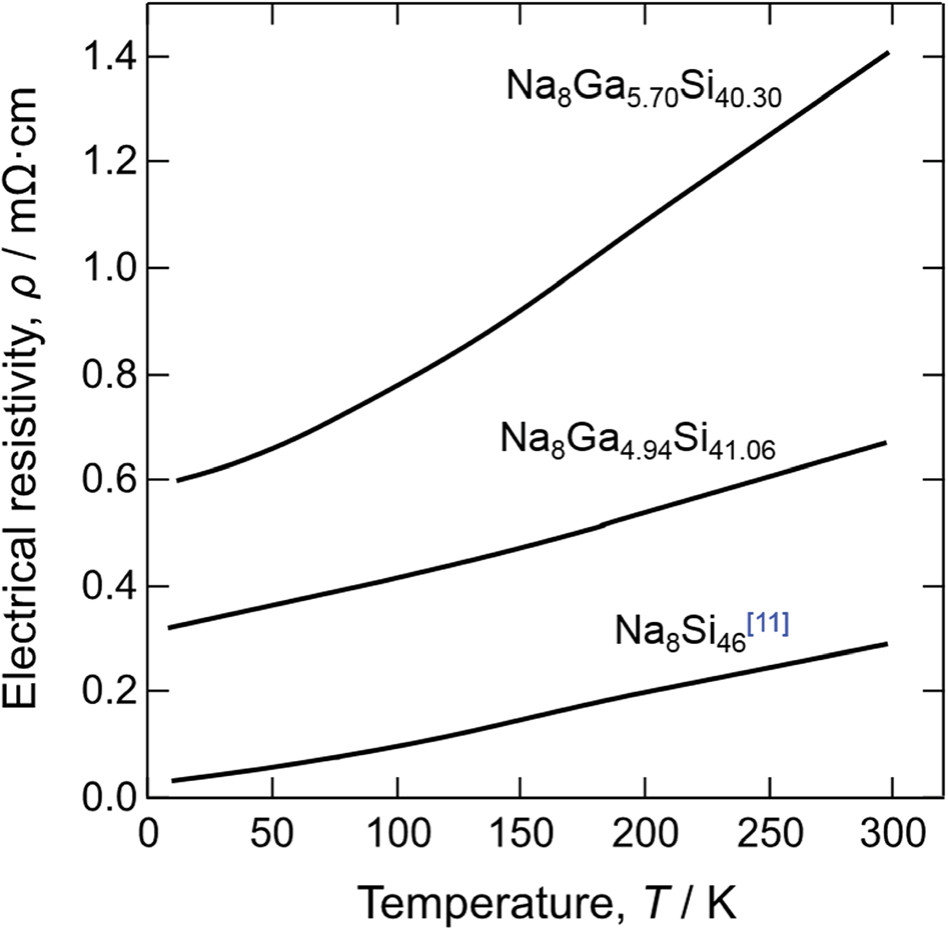
Temperature dependence of electrical resistivity for single crystals of Na_8_Ga_5.70_Si_40.30_, Na_8_Ga_4.94_Si_41.06_, and Na_8_Si_46_.^11^

The electrical resistivities measured for crystal 1 (Na_8_Ga_5.70_Si_40.30_) and crystal 5 (Na_8_Ga_4.94_Si_41.06_) together with the resistivity of Na_8_Si_46_ (ref. 11) are shown against the temperature in Fig. 7. These crystals showed metallic behavior. The electrical resistivity decreased as the Ga content decreased, and those of Na_8_Ga_5.70_Si_40.30_ and Na_8_Ga_4.94_Si_41.06_ at 300 K were 1.40 and 0.72 mΩ cm, respectively. These values were greater than the resistivity of Na_8_Si_46_ (0.24 mΩ cm at 300 K (ref. 11)). In the case of Na_8_Si_46_, most electrons supplied from Na to the Si cage remain in the conduction band and contribute to the metallic conduction. When a part of tetravalent Si atoms is replaced by trivalent Ga atoms, some electrons from the Na atoms are present in the valence band owing to the decrease in the valence electrons through Ga substitution.

A_8_Ga_8_Si_38_ (A = K, Rb, Cs) synthesized by Sui *et al.*^17^ are semiconductors, exhibiting a decrease in the electrical resistivities with an increase in temperature. This is attributed to the fact that the number of electrons provided by A atoms is the same as the number of valence electrons decreased through substitution of Ga for Si atoms in the cage, which fulfills the octet condition. The first-principle calculation by Imai *et al.*^13^ predicted that Na_8_Ga_8_Si_38_ is a semiconductor with a band gap of 0.448 eV. As the maximum Ga content *x* = 5.70(7) of Na_8_Ga_*x*_Si_46−*x*_ synthesized in the present study is less than 8, the surplus electrons from Na remain in the conduction band and contribute to the metallic conduction as in the case of Na_8_Si_46_. Similar metallic conduction was also reported for K_8_Al_7_Si_39_.^26,27^ Dong *et al.* suggested that the Al content of *x* would be slightly less than 8 for Na_8_Al_8_Si_38_, judging from the weak temperature dependence of electrical conductivity with a small activation energy.^16^

## 4. Conclusions

Single crystals of novel Na–Ga–Si ternary type-I clathrates were successfully grown by evaporating Na from a Na–Ga–Si or Na–Ga–Si–Sn melt. A single crystal of Na_8_Ga_5.70_Si_40.30_ of size 2.9 mm was obtained from the Na–Ga–Si melt by heating at 773 K for 21 h. The Ga content *x* of Na_8_Ga_*x*_Si_46−*x*_ single crystals grown from the Na–Ga–Si–Sn melt decreased from 5.52 to 4.94 with the increase in the heating temperature from 723 to 873 K. The size of a Na_8_Ga_4.94_Si_41.06_ single crystal grown from the Na–Ga–Si–Sn melt at 873 K was 3.7 mm. The metallic temperature dependences of the electric resistivities expected from the formula of Na_8_Ga_*x*_Si_46−*x*_ (*x* = 5.70, 4.94) were confirmed for the single crystals.

## Conflicts of interest

There are no conflicts to declare.

## Supplementary Material

RA-008-C8RA07971D-s001

RA-008-C8RA07971D-s002

## References

[cit1] Kasper J. S., Hagenmuller P., Pouchard M., Cros C. (1965). Science.

[cit2] Adams G. B., O'Keeffe M., Demkov A. A., Sankey O. F., Huang Y.-M. (1994). Phys. Rev. B.

[cit3] Dolyniuk J.-A., Owens-Baird B., Wang J., Zaikina J. V., Kovnir K. (2016). Mater. Sci. Eng. R Rep..

[cit4] Kawaji H., Horie H., Yamanaka S., Ishikawa M. (1995). Phys. Rev. Lett..

[cit5] Morito H., Yamada T., Ikeda T., Yamane H. (2009). J. Alloys Compd..

[cit6] Pouchard C. C. M. (2009). C. R. Chim..

[cit7] Horie H., Kikudome T., Teramura K., Yamanaka S. (2009). J. Solid State Chem..

[cit8] Beekman M., Baiitinger M., Borrmann H., Schnelle W., Meier K., Nolas G. S., Grin Y. (2009). J. Am. Chem. Soc..

[cit9] Stefanoski S., Beekman M., Woung-Ng W., Zavalij P., Nolas G. S. (2011). Chem. Mater..

[cit10] Stefanoski S., Blosser M. C., Nolas G. S. (2013). Cryst. Growth Des..

[cit11] Morito H., Shimoda M., Yamane H. (2016). J. Cryst. Growth.

[cit12] Morito H., Shimoda M., Yamane H., Fujiwara K. (2018). Cryst. Growth Des..

[cit13] Imai Y., Imai M. (2011). J. Alloys Compd..

[cit14] Nakamura K., Yamada S., Ohnuma T. (2013). Mater. Trans..

[cit15] Baran V., Senyshyn A., Karttunen A. J., Fischer A., Scherer W., Raudaschl-Sieber G., Fssler T. F. (2014). Chem.–Eur. J..

[cit16] Dong Y., Chai P., Beekman M., Zeng X., Tritt T. M., Nolas G. S. (2015). Inorg. Chem..

[cit17] Sui F., He H., Bobev S., Zhao J., Osterloh F. E., Kauzlarich S. M. (2015). Chem. Mater..

[cit18] Westerbgays W., Schuster H.-U. (1977). Z. Naturforsch.

[cit19] Bruker AXS , Instrument Service v4.2.7, APEX2, SADABS, SAINT-Plus & XPREP, Bruker AXS Inc., Madison, Wisconsin, USA, 2014

[cit20] Sheldrick G. M. (2015). Acta Crystallogr..

[cit21] Momma K., Izumi F. (2011). J. Appl. Crystallogr..

[cit22] Terauchi M., Koshiya S., Satoh F., Takahashi H., Handa N., Murano T., Koike M., Imazono T., Koeda M., Nagano T., Sasai H., Oue Y., Yonezawa Z., Kuramoto S. (2014). Microsc. Microanal..

[cit23] Terauchi M., Takahashi H., Takamura M., Murano T., Koike M., Imazono T., Nagano T., Sasai H., Koeda M. (2016). Microsc. Microanal..

[cit24] Moewes A., Kurmaev E. Z., Tse J. S., Geshi M., Ferguson M. J., Trofimova V. A., Yarmoshenko Y. M. (2002). Phys. Rev. B.

[cit25] Hubbard C. R., Swanson H. E., Mauer F. A. (1975). J. Appl. Crystallogr..

[cit26] Imai M., Singh S. K., Nishio M., Yamada T., Yamane H. (2015). Jpn. J. Appl. Phys..

[cit27] Singh S. K., Mochiku T., Ibuka S., Isoda Y., Hoshikawa A., Ishigaki T., Imai M. (2015). Jpn. J. Appl. Phys..

