# Supplementary material for: Na–Ga–Si type-I clathrate single crystals grown via Na evaporation using Na–Ga and Na–Ga–Sn fluxes
Source: RSC Adv. 2018 Dec 4;8(71):40505–10. doi: 10.1039/c8ra07971d (PMC9091382; doi:10.1039/c8ra07971d)
Supplement: RA-008-C8RA07971D-s001 [file RA-008-C8RA07971D-s001.pdf]

Electronic Supplementary Material (ESI) for RSC Advances.
This journal is © The Royal Society of Chemistry 2018

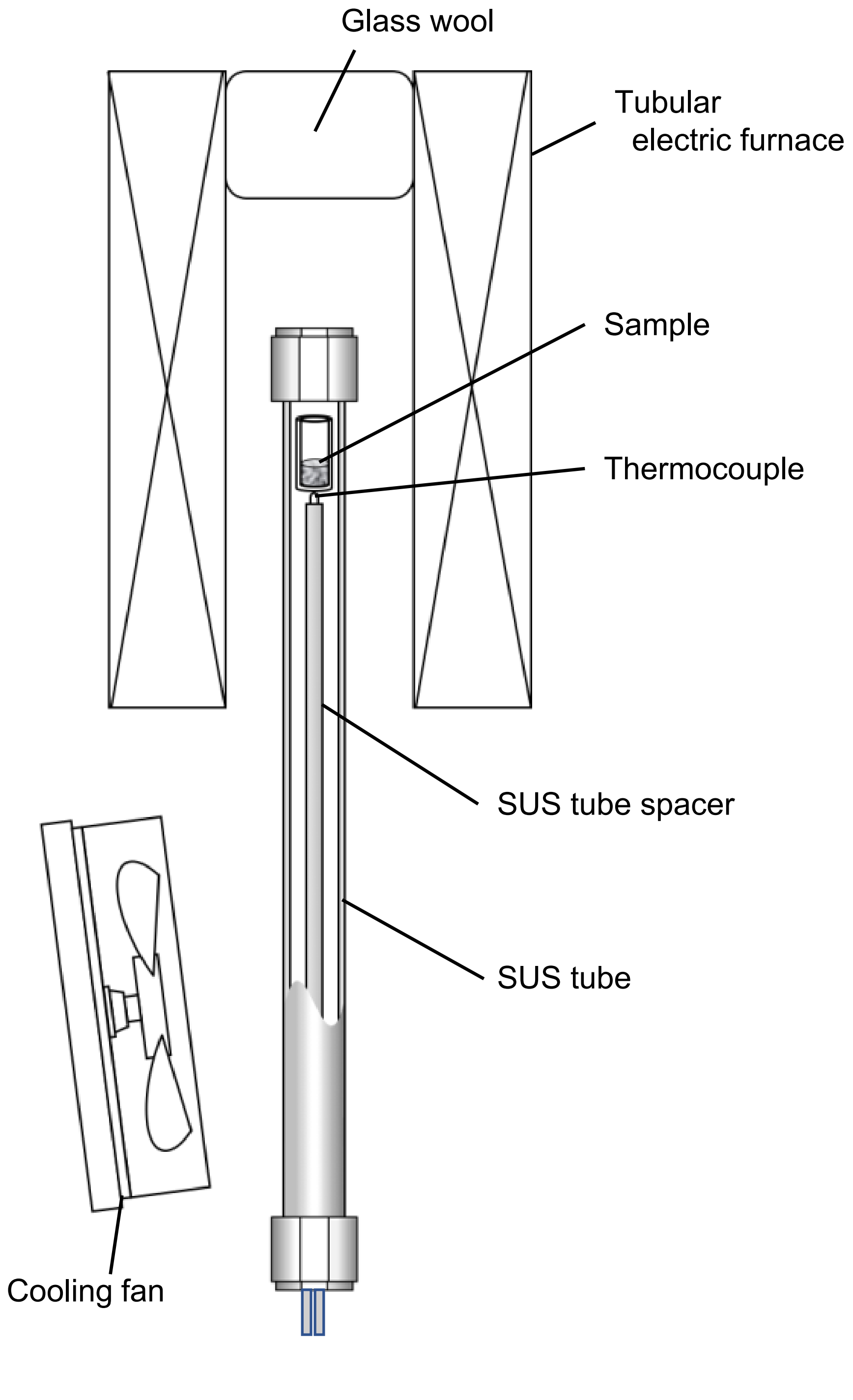

Figure S1. Schematic illustration of a stainless steel (SUS) container and a tubular electric furnace.

Table S1 Atomic coordinates and isotropic and equivalent isotropic displacement parameter (U_{eq} / Å^2) for **Crystals 1−5**.

atom	site	occupancy	*x*	*y*	*z*	U_{eq}
**Crystal 1** ($Na_8Ga_{5.70(7)}Si_{40.30}$)						
Na1	6*d*	1	1/4	1/2	0	0.0678(7)
Na2	2*a*	1	0	0	0	0.0235(6)
Si/Ga1	24*k*	0.945 / 0.055(2)	0	0.30544(4)	0.11644(4)	0.00831(16)
Si2	16*i*	1	0.18400(3)	*x*	*x*	0.0075(2)
Si/Ga3	6*c*	0.270 / 0.730(4)	1/4	0	1/2	0.00801(17)
**Crystal 2** ($Na_8Ga_{5.52(7)}Si_{40.48}$)						
Na1	6*d*	1	1/4	1/2	0	0.0664(7)
Na2	2*a*	1	0	0	0	0.0238(6)
Si/Ga1	24*k*	0.947 / 0.053(2)	0	0.30552(4)	0.11645(4)	0.00861(16)
Si2	16*i*	1	0.18405(3)	*x*	*x*	0.0081(2)
Si/Ga3	6*c*	0.292 / 0.708(4)	1/4	0	1/2	0.00852(17)
**Crystal 3** ($Na_8Ga_{5.39(11)}Si_{40.61}$)						
Na1	6*d*	1	1/4	1/2	0	0.0662(10)
Na2	2*a*	1	0	0	0	0.0238(9)
Si/Ga1	24*k*	0.947 / 0.053(3)	0	0.30560(5)	0.11650(5)	0.0092(2)
Si2	16*i*	1	0.18407(4)	*x*	*x*	0.0086(3)
Si/Ga3	6*c*	0.315 / 0.685(6)	1/4	0	1/2	0.0090(2)
**Crystal 4** ($Na_8Ga_{5.06(7)}Si_{40.94}$)						
Na1	6*d*	1	1/4	1/2	0	0.0636(6)
Na2	2*a*	1	0	0	0	0.0236(5)
Si/Ga1	24*k*	0.955 / 0.045(2)	0	0.30566(3)	0.11651(3)	0.00825(15)
Si2	16*i*	1	0.18407(2)	*x*	*x*	0.00793(18)
Si/Ga3	6*c*	0.336 / 0.664(4)	1/4	0	1/2	0.00809(16)
**Crystal 5** ($Na_8Ga_{4.94(6)}Si_{41.06}$)						
Na1	6*d*	1	1/4	1/2	0	0.0619(6)
Na2	2*a*	1	0	0	0	0.0229(5)
Si/Ga1	24*k*	0.9561 / 0.0439(18)	0	0.30573(3)	0.11652(3)	0.00825(14)
Si2	16*i*	1	0.18408(2)	*x*	*x*	0.00778(17)
Si/Ga3	6*c*	0.352 / 0.648(3)	1/4	0	1/2	0.00807(15)

Table S2 Atomic coordinates and isotropic and equivalent isotropic displacement parameter (U_{ij} / Å^2) for **Crystals 1−5**.

atom	U_{11}	U_{22}	U_{33}	U_{23}	U_{13}	U_{12}
**Crystal 1** ($Na_8Ga_{5.70(7)}Si_{40.30}$)						
Na1	0.0459(13)	0.0787(11)	$= U_{22}$	0	0	0
Na2	0.0235(6)	$= U_{11}$	$= U_{11}$	0	0	0
Si / Ga1	0.0083(2)	0.0090(2)	0.0077(2)	−0.00011(14)	0	0
Si2	0.0075(2)	$= U_{11}$	$= U_{11}$	−0.00038(10)	$= U_{23}$	$= U_{23}$
Si / Ga3	0.0089(2)	0.00757(18)	$= U_{22}$	0	0	0
**Crystal 2** ($Na_8Ga_{5.52(7)}Si_{40.48}$)						
Na1	0.0462(14)	0.0765(11)	$=U_{22}$	0	0	0
Na2	0.0238(6)	$=U_{11}$	$=U_{11}$	0	0	0
Si1 / Ga1	0.0086(2)	0.0091(2)	0.0082(2)	−0.00014(12)	0	0
Si2	0.0081(2)	$=U_{11}$	$=U_{11}$	−0.00031(9)	$=U_{23}$	$=U_{23}$
Si3 / Ga3	0.0096(2)	0.00799(19)	$=U_{22}$	0	0	0
**Crystal 3** ($Na_8Ga_{5.39(11)}Si_{40.61}$)						
Na1	0.046(2)	0.0765(16)	$=U_{22}$	0	0	0
Na2	0.0238(9)	$=U_{11}$	$=U_{11}$	0	0	0
Si1 / Ga1	0.0091(3)	0.0098(3)	0.0087(3)	0.0000(2)	0	0
Si2	0.0086(3)	$=U_{11}$	$=U_{11}$	−0.00044(15)	$=U_{23}$	$=U_{23}$
Si3 / Ga3	0.0097(4)	0.0087(3)	$=U_{22}$	0	0	0
**Crystal 4** ($Na_8Ga_{5.06(7)}Si_{40.94}$)						
Na1	0.0451(12)	0.0728(9)	$=U_{22}$	0	0	0
Na2	0.0236(5)	$=U_{11}$	$=U_{11}$	0	0	0
Si1 / Ga1	0.0081(2)	0.0087(2)	0.0079(2)	−0.00022(12)	0	0
Si2	0.00793(18)	$=U_{11}$	$=U_{11}$	−0.00041(9)	$=U_{23}$	$=U_{23}$
Si3 / Ga3	0.0091(2)	0.00757(17)	$=U_{22}$	0	0	0
**Crystal 5** ($Na_8Ga_{4.94(6)}Si_{41.06}$)						
Na1	0.0428(11)	0.0715(9)	$=U_{22}$	0	0	0
Na2	0.0229(5)	$=U_{11}$	$=U_{11}$	0	0	0
Si / Ga1	0.00813(19)	0.00885(19)	0.00776(19)	−0.00024(12)	0	0
Si2	0.00778(17)	$=U_{11}$	$=U_{11}$	−0.00035(9)	$=U_{23}$	$=U_{23}$
Si / Ga3	0.0089(2)	0.00768(16)	$=U_{22}$	0	0	0

Table S3 Atomic distances (Å) for **Crystals 1−5**.

crystal	1	2	3	4	5
composition	$Na_8Ga_{5.70(7)}Si_{40.30}$	$Na_8Ga_{5.52(7)}Si_{40.48}$	$Na_8Ga_{5.39(11)}Si_{40.61}$	$Na_8Ga_{5.06(7)}Si_{40.94}$	$Na_8Ga_{4.94(6)}Si_{41.06}$
$[Si/Ga]_{24}$ cage					
Na1-Si/Ga1	3.4853(3)	3.4830(3)	3.4827(4)	3.4783(2)	3.4755(2)
Na1-Si2	3.8371(4)	3.8348(4)	3.8347(5)	3.8302(3)	3.8275(3)
Na1-Si/Ga3	3.6510(1)	3.6490(1)	3.6490(1)	3.6448(1)	3.6423(1)
Si/Ga1-Si/Ga1	2.4049(7)	2.4037(8)	2.4048(11)	2.4022(7)	2.4007(6)
Si/Ga1-Si2	2.3811(3)	2.3805(3)	2.3809(4)	2.3785(2)	2.3772(2)
Si/Ga1-Si/Ga3	2.4370(4)	2.4350(4)	2.4339(6)	2.4306(3)	2.4283(3)
Si2-Si2	2.3610(10)	2.3580(10)	2.3575(15)	2.3544(9)	2.3526(8)
Si-Si ave.	2.4001(5)	2.3987(5)	2.3986(7)	2.3958(4)	2.3941(3)
$[Si/Ga]_{20}$ cage					
Na2-Si/Ga1	3.3756(4)	3.3745(4)	3.3756(6)	3.3722(3)	3.3706(3)
Na2-Si2	3.2910(5)	3.2901(5)	3.2904(7)	3.2867(4)	3.2846(4)
Si/Ga1-Si/Ga1	2.4049(7)	2.4037(8)	2.4048(11)	2.4022(7)	2.4007(6)
Si/Ga1-Si2	2.3811(3)	2.3805(3)	2.3809(4)	2.3785(2)	2.3772(2)
Si-Si ave.	2.3859(4)	2.3851(4)	2.3857(5)	2.3832(3)	2.3819(3)
